# Expert Motor Synergies Emerge Predominantly Offline During Early Skill Learning

**DOI:** 10.64898/2026.02.24.707000

**Published:** 2026-02-25

**Authors:** William Kistler, Rawan Fakhreddine, Giselle R Rodriguez, Margaret Hayward, Ethan R. Buch, Sven Bestmann, Leonardo G. Cohen

**Affiliations:** 1 Human Cortical Physiology and Neurorehabilitation Section, National Institute of Neurological Disorders and Stroke, NIH, Bethesda, MD, USA; 2 Department of Clinical and Movement Neuroscience, UCL Queen Square Institute of Neurology, London, United Kingdom; 3 Department of Imaging Neuroscience, UCL Queen Square Institute of Neurology, London, United Kingdom

## Abstract

Everyday skilled actions depend on the formation of coordinated motor synergies that integrate multiple effectors into stable, low-dimensional control units. Although initial practice of a new skill leads to rapid performance improvements, it is unclear whether the underlying movement kinematics reorganize on a similar timescale and in a way that directly relates to these gains. It also remains uncertain whether such reorganization occurs mainly during active practice or instead during brief rest breaks.

Here, we tracked the temporal evolution of multi-digit synergy formation during early learning of a naturalistic keypress skill. Initial practice rapidly sculpted the motor repertoire toward higher-order, temporally compressed and overlapping multi-digit synergies. Highly stable synergies emerged after only minutes of practice and continued to be expressed across two training days. Notably, these expert synergies were primarily shaped during brief rest breaks and robustly predicted individual skill proficiency. Across learning, distinct synergy subtypes were evident, differing in their prevalence. Rarely expressed synergies reflected transient novice patterns, synergies expressed at intermediate levels indexed exploratory and trial-initiation strategies, and highly expressed synergies emerged later to dominate performance, reflecting the consolidation and expansion of skilled motor control.

Together, these findings indicate that skilled performance is supported by the early formation of a compact repertoire of expert multi-digit synergies that emerge preferentially across rest periods and predict subsequent skill gains. They further raise the hypothesis that explicitly training such expert synergies—alongside task goals—could enhance learning in domains such as the arts, sport, and neurorehabilitation.

## Introduction

Activities of daily living—from walking and handwriting to athletic play—depend on the precise orchestration of temporally ordered motor sequences [1–4]. Fluency develops as the motor system learns to coordinate fingers in new ways. These task-specific movement patterns link motions within and across digits so that they work together to meet the demands of the object, the required force, timing, and accuracy [5, 6]. With practice, discrete keypresses become coarticulated into coordinated multi-digit synergies, reducing effective control dimensionality and stabilizing execution, thereby optimizing performance across skills such as grasping [7–9], typing [10], finger spelling[11], and haptic exploration [12]. Studying how motor synergies emerge and reorganize provides a direct window into the neuromotor mechanisms that support the acquisition, refinement, and generalization of skilled actions [13].

Early learning of naturalistic skills is characterized by prominent initial performance improvements [14–16]. Over the course of learning, skill performance fluctuates during practice and brief rest periods as a result of the dynamic interplay between performance-enhancing processes—such as learning—and performance-limiting mechanisms, including motor or cognitive fatigue (sometimes referred to collectively as reactive inhibition). However, how these competing processes are expressed in the kinematic signatures that drive the transition from novice movement patterns to expert multi-digit synergies—particularly during the earliest phases of skill acquisition—is not known. Early gains in skill may arise from several, non-mutually exclusive, kinematic adjustments: faster execution [17], greater temporal overlap among digit movements (coarticulation) [18–20], reparameterization of velocity profiles [21], improved spatial accuracy [22] or their combination. In the temporal domain, the formation of multi-digit synergies characteristic of expertise could unfold rapidly—on the order of seconds—or accrue more slowly over minutes to hours [23, 24]. Such reorganization may also proceed during brief rest intervals [25] or instead manifest primarily during active practice, in parallel with expansion of memory capacity [26]. This pattern could suggest a dissociation between the time course of behavioral improvement and the optimization of underlying kinematics. In addition, learning processes such as exploration, prospective planning [27], and expansion of memory capacity [26] may be distinctly expressed in the transition from novice patterns to expert multi-digit synergies.

Here, we addressed these questions by characterizing the temporal evolution of multi-digit kinematics—including the emergence and reorganization of skill-specific synergies—during the early learning of a naturalistic motor skill. We show that complex, multi-digit synergies emerge within seconds of practice and are predominantly recruited during early rest periods, with no evidence that their formation or stability is disrupted by fatigue. These synergies, composed of small, rapid, and overlapping digit movements, developed gradually over initial training trials and reliably predicted subsequent skill gains. Notably, these multi-digit coordination patterns were maintained on the following day, underscoring the stability of their neural representations in supporting the newly acquired skill.

## Results

**FIGURE 1:**
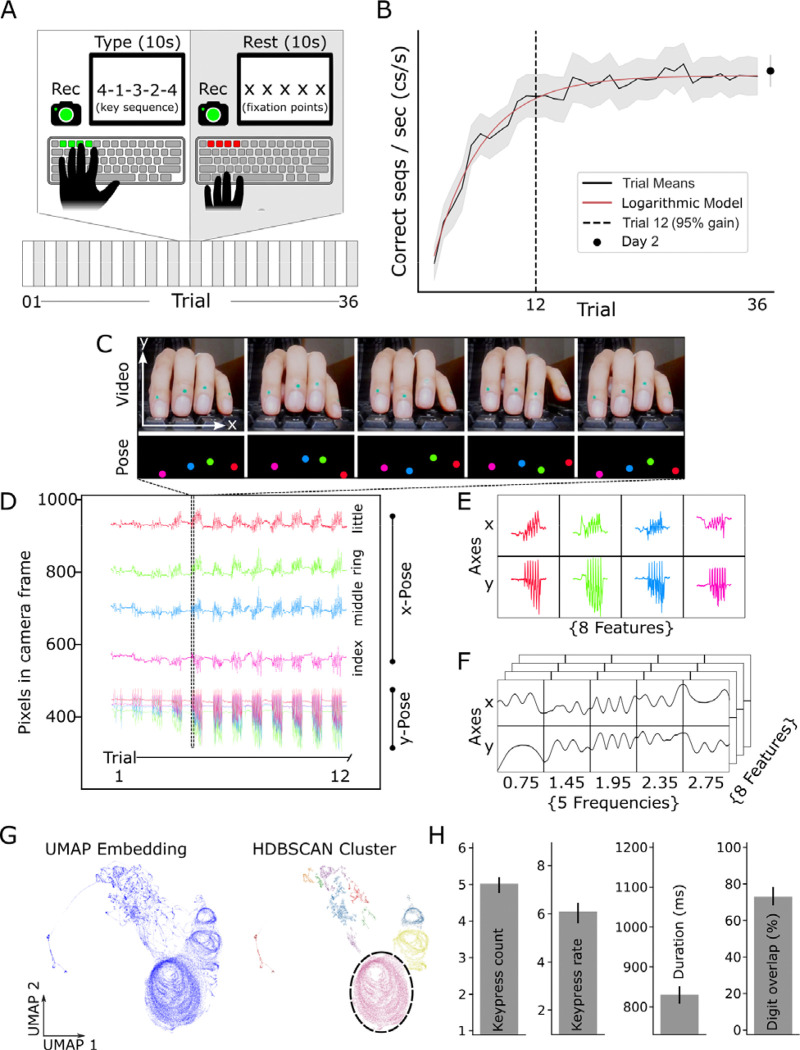
Data analysis (A) Subjects learned a motor sequence over 36 practice trials in a single training session [30, 74]. Each trial alternated between 10 s training (sequence typing) and 10 s rest periods. (B) Skill was quantified as the rate of correctly typed sequences per second. Early learning, the training period during which 95% of maximum performance was reached, developed within the initial 12 trials (mean ± 95% CI; logarithmic model fit shown [15]). The dashed vertical line indicates the trial by which 95% of maximum performance was reached. The black dot shows performance at the follow-up test on Day 2. (C) High-definition videos captured from a frontal viewpoint (top) were analysed using markerless pose estimation to track digit positions on each frame (bottom). (D) For a representative participant, digit displacement trajectories along the lateral (x) and vertical (y) axes are shown across the first 12 trials, plotted as raw pixel coordinates derived from DeepLabCut. As practice progressed, the amplitude of digit movements increased, becoming especially pronounced by trial 12. (E) Digit trajectories during a single practice trial (10sec) in a representative subject yielding 8 features (four digits × two axes). Digit coding as in D. (F) The time-domain signals shown in panel E were subsequently transformed into the frequency domain using a Fast Fourier Transform (FFT) and then convolved with Morlet wavelets at five movement-relevant frequencies that are characteristically expressed during digit movements [37, 38]. (G) *Left*: Wavelet-transformed digit movements (panel F) were embedded into a two-dimensional space using Uniform Manifold Approximation and Projection (UMAP) shown here for a single participant across the full 36-trial practice session. Each point corresponds to one video frame sampled at 120 Hz. *Right*: Clustering performed on the UMAP embedding using Hierarchical Density-Based Spatial Clustering of Applications with Noise (HDBSCAN) to identify density-defined clusters (behavioural synergies) (H) The kinematic properties of a representative synergy cluster (circled in panel G, right) are shown, including the number of keypresses (5 kp), keypress rate (6 kp/s), duration (840 ms), and the proportion of temporal digit overlap within the synergy (75%).

We examined kinematic features of early skill acquisition in twenty healthy adults (12 female; mean age 30 years) trained on a well-characterized motor sequence task [14, 28–31]. Subjects were tasked with repetitively typing a 5-item keypress sequence displayed on the screen (4–1-3–2-4) as quickly and accurately as possible with 4 digits (index, middle, ring and little fingers) of their left, non-dominant hand. Each trial comprised 10 seconds of practice followed by 10 seconds of rest, yielding a session of approximately 15 minutes length (Fig 1A). Motor skill was indexed by the rate of correct keypresses per second (kp/s) [32, 33]. The early learning period encompassed the training trials during which 95% of maximum performance was reached [15, 33], which, at the group level, occurred by trial 12 (Fig 1B).

### Characterization of movement synergies

Digit trajectories were recorded at 120 Hz with a front-facing high-speed camera (Fig 1C, **top**). Markerless pose estimation [34–36] tracked distal interphalangeal joints, yielding ~42,500 x–y frames per participant across the full practice session. The x-axis primarily captured in-plane lateral motion (adduction–abduction), whereas the y-axis reflected vertical flexion–extension perpendicular to the keyboard (Fig 1C–E). For each practice trial, digit trajectories were represented as multivariate time series comprising eight features (four digits × two spatial axes; Fig. 1E). Time series were decomposed with complex Morlet wavelets centred on five movement-relevant frequencies [37, 38] (following FFT), providing time-resolved amplitude and phase (Fig 1F). For each time point, we formed feature vectors from amplitude and phase across digits and frequencies, embedded them into two dimensions with UMAP (see Methods) using all practice time points per subject, and applied HDBSCAN (see Methods) to identify density-defined clusters (behavioural synergies) while labelling low-density points as noise (Fig 1G, Methods). To quantify training-dependent changes in behavioural synergies, we computed the Jensen–Shannon divergence (JSD) index between HDBSCAN-derived synergy maps (Suppl Fig. 1). Kinematic features of individual synergies—including keypress count, keypress rate, duration, and inter-digit overlap—were obtained by projecting cluster labels back into the original feature space (Fig 1H).

### Emergence of multi-digit synergies during early skill learning

**FIGURE 2:**
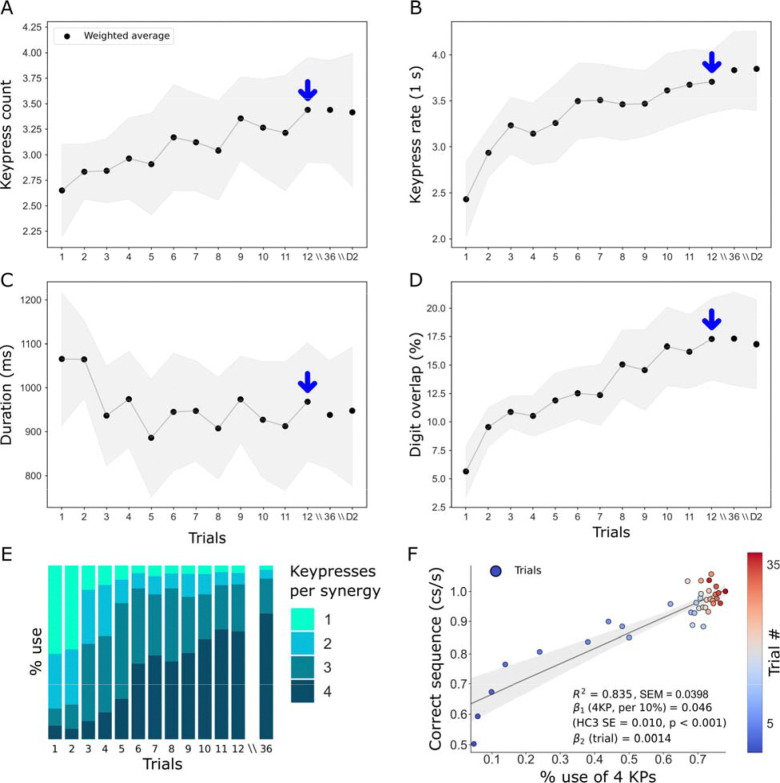
Kinematic properties of motor synergies. **(A)** Mean keypress count (kp/synergy) rose steadily across the early practice trials, attaining an asymptote near trial 12 (blue arrow), after which it plateaued. **(B)** The mean keypress rate (kp/second), normalized by synergy duration, exhibited progressive increases across practice, reflecting steady gains in typing speed. These improvements continued through trial 36 on Day 1 and were maintained at the Day 2 follow-up. **(C)** The mean duration of individual synergies within each trial decreased rapidly during early practice. **(D)** Digit overlap—defined as the mean proportion of the synergy interval during which multiple digits move concurrently—increased progressively with training and remained elevated on Day 2. Blue arrows in panels A–D indicate trial 12, the point at which participants had achieved approximately 95% of their maximum performance, marking the end of early learning [15]. **(E)** Stacked bar plots show the proportional deployment of synergies comprising one, two, three, or four keypresses across training. Note that with practice, participants gradually reweighted their repertoire from early reliance on single/double keypress synergies (light blues) to preferential use of synergies containing three and four keypresses (darker blues). **(F)** Linear regression was used to quantify the relationship between the proportion of four-keypress synergies and skill (cs/s), with each point representing a single trial and color-coded by Day 1 trial number (1–36). Across practice, increased reliance on four-keypress synergies closely paralleled improvements in skill (R^2^ = 0.83, p < 0.001; SEM = 0.0398).

Training was associated with a progressive increase in both the mean keypress count per synergy (Fig 2A) and the overall typing rate (Fig 2B). In parallel, the duration of individual synergies decreased (Fig. 2C), while temporal overlap across digit movements increased (Fig. 2D), collectively indicating a shift from predominantly isolated single-digit synergies toward more integrated, multi-digit, and coarticulated synergies. Indeed, from Trials 1 to 12, 1-keypress synergies fell to near extinction, while 2- and 3-keypress synergies rose steeply early on and then declined once learning plateaud. In contrast, 4- keypress synergies increased progressively during early learning and then stabilized (Fig 2E, Suppl Fig 2). Critically, the % use of 4- keypress synergies correlated with the magnitude of skill learning (R^2^ = 0.83, p < 0.001; SEM = 0.0398; Fig 2F). Collectively, these findings indicate that early practice rapidly prunes the motor repertoire toward temporally compressed, overlapping, high-order multi-digit synergies, whose stabilized dominance from approximately trial 12 onward predicts the magnitude of the acquired skill.

### No detectable impact of fatigue on early-learning kinematics.

We next asked whether accumulating fatigue with continued practice [39] might influence this kinematic transformation. Fatigue-related motor slowing [40] could result in the progressive attrition of multi-digit synergies—either within individual 10-s practice trials or cumulatively across the 36-trial session. Contrary to these predictions, once multi-digit synergies were established by the end of early learning, their expression remained stable, showing no decline within individual trials at the end of training (when fatigue could be expected, Suppl. Fig. 3) or across the entire training session (Fig 2E). We further asked whether hypothetical end-of-Day 1 fatigue-induced deterioration in multi-digit synergy use recover after overnight sleep. To this end, we compared 4-keypress synergy use at the end of Day 1—when fatigue would be most likely—with that at Day 2 retest. If fatigue had degraded synergy kinematics by the end of Day 1, we would expect a Day 2 rebound (increased multi-digit synergy use), yet 4-keypress synergy expression was comparable and did not rebound across days (Table 3). Thus, early learning of a naturalistic skill proceeds without detectable fatigue-related disruption of multi-digit synergies.

### Higher-Order Multi-Digit Synergies Emerge Across Rest Intervals Interleaved Practice

Prior work indicates that early gains in challenging, naturalistic motor skills may accrue across brief rest intervals (“micro-offline”) to a larger extent than during active practice (“micro-online”) [15]. Here, we replicated this observation. On average, micro-online changes were negligible (relative to 0, no change, mean = −0.297 ± 0.325 cs/s; two-tailed one-sample t-test, t_19_ = −0.53, p = 0.60), whereas micro-offline gains were robust (mean = 1.006 ± 0.317 cs/s; two-tailed one-sample t-test, t_19_ = 4.61, p = 1.92 × 10^−^). Micro-offline gains significantly exceeded micro-online changes (paired t-test, t_19_ = 3.68, p = 0.0016) and were statistically indistinguishable from total early learning (mean = 0.709 ± 0.055 cs/s; paired t-test, t_19_ = 1.86, p = 0.079).

**FIGURE 3.**
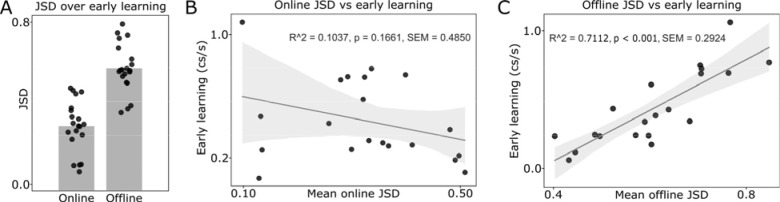
HDBSCAN-derived synergy maps reorganize more during rest than during practice in early learning. **(A)** During early learning (trials 1–12), Jensen–Shannon divergence (JSD) between consecutive synergy maps was greater across rest intervals than across practice intervals, indicating stronger reorganization during brief pauses. **(B)** Changes in synergy maps across practice intervals (online JSD) did not reliably predict early learning (R^2^ = 0.104, p = 0.166, SEM = 0.485). **(C)** By contrast, changes in synergy maps across rest intervals (offline JSD) strongly correlated with early learning (R^2^ = 0.711, p < 0.001, SEM = 0.292), suggesting that key aspects of synergy reconfiguration supporting early performance gains occur predominantly offline.

Changes in HDBSCAN-derived synergy maps (Fig 1G) developed predominantly across rest (mean = 0.596; SEM ±0.026) rather than practice (mean = .316; SEM ±0.028) intervals (Fig 3A). JSD divergence across rest intervals robustly predicted early learning (R^2^ = 0.711, p < 0.001; Fig. 3C), whereas JSD divergence during practice bouts did not (R^2^ = 0.104, p = 0.166; Fig. 3B).

Together with the absence of fatigue effects on early-learning kinematics of a challenging naturalistic skill, these findings support the view that the transition to higher-order, multi-digit coordination is driven predominantly by micro-offline processes unfolding across brief rest intervals.

### Synergies subserving early learning of a naturalistic skill

**Figure 4:**
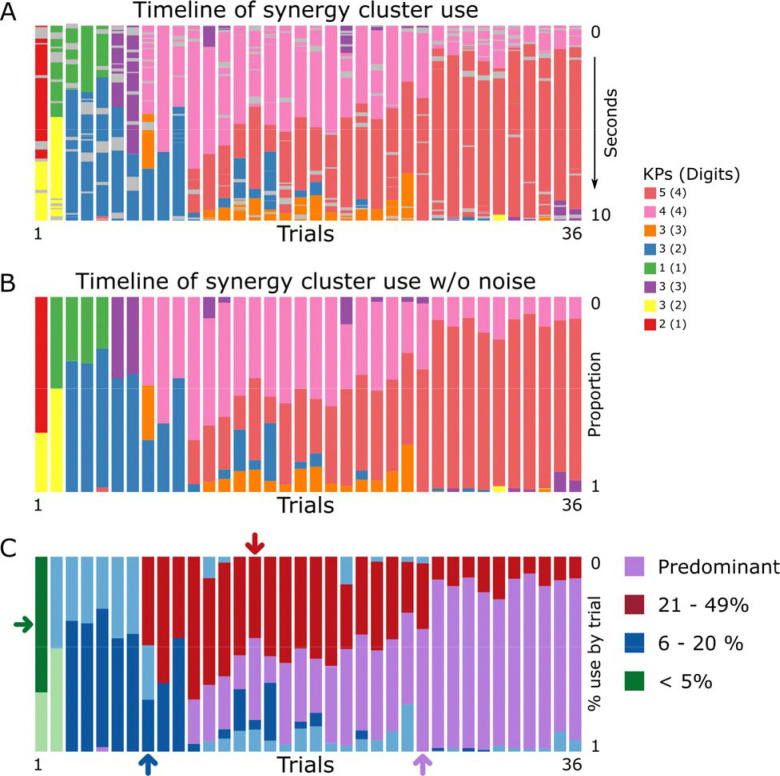
Emergence of expert synergies during early learning in a representative subject. **(A)** Bar plot depicting the temporal distribution of synergy clusters across each 10-s practice interval over 36 trials in the same representative participant depicted in Fig 1G. Gray segments mark epochs within a trial in which no stable synergies were identified (noise). Across training, a single predominant synergy (purple) emerges and progressively dominates behavior, illustrating the stabilization of a preferred coordination pattern with practice. **(B):** Timeline of synergy cluster occurrences (excluding noise), normalized to trial duration (0–1) for the same representative participant. This bar plot parallels panel A but omits gray noise epochs, showing only the sequence of identified synergies across each 10-s practice interval (n = 36 trials). This noise-free, time-normalized representation was used for all subsequent JSD-based statistical analyses (Suppl. Fig. 1). The number of keypresses and digits per synergy is indicated on the right. Early in training, synergies predominantly comprise single or double digits, whereas later-emerging, dominant synergies recruit multiple digits, reflecting the development of higher-order coordination. **(C)** Synergies were subsequently classified according to their overall percentage of use across all training trials (>50%, 21–49%, 6–20%, and <5%). Several distinct temporal profiles emerged in this subject. Some synergies were expressed almost exclusively in the very first trial(s) (e.g., green arrow, “novice”), whereas others appeared transiently during mid-practice (e.g., dark blue arrow, “exploratory”). Certain synergies were expressed predominantly at trial onset as performance approached asymptote (e.g., red arrow, “trial initiation”), while others emerged around mid-training and became increasingly dominant toward the end of practice (e.g., purple arrow, “expert”). This qualitative pattern, illustrated here for a single participant, was also observed at the group level (Fig. 5).

**Figure 5.**
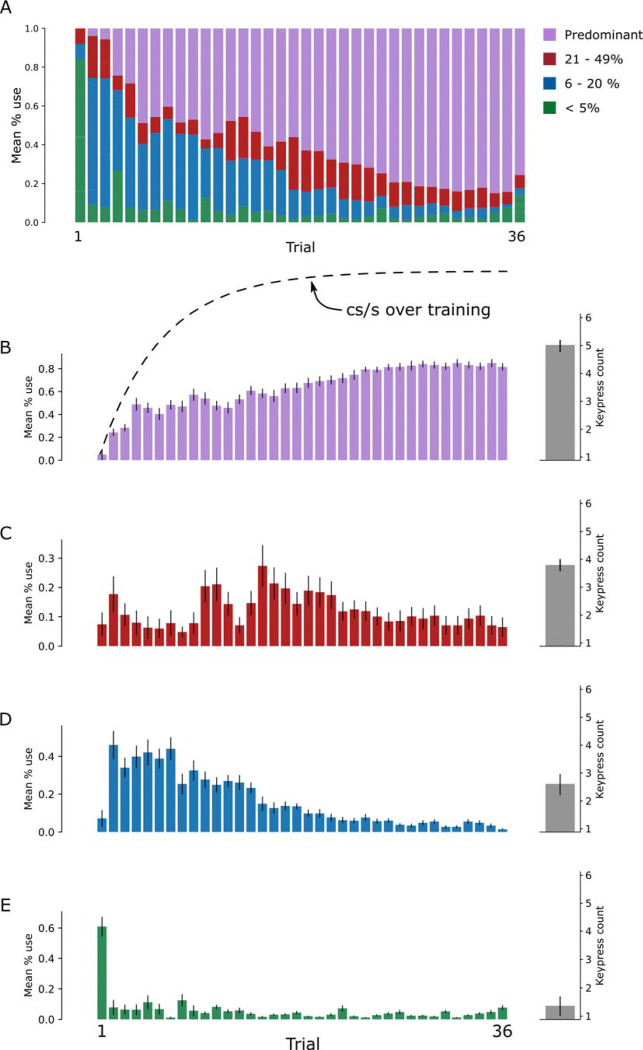
Synergy types emerging during early learning of a challenging naturalistic skill. **(A)** Synergies were classified according to their overall percentage of use across all training trials (>50%, 21–49%, 6–20%, <5%). Group-level stacked bars show the average percent use of each synergy class across trials (1–36). **(B–E)** Group-averaged percent use (± SEM) for each synergy type across trials and subjects. The learning curve is overlaid in panel **B** (dashed line) for reference. Gray bars to the right of each panel indicate the mean number of keypresses encompassed by each synergy type (± SEM). At the group level, the pattern observed in the single-subject example (Fig. 4) is preserved: predominant 5-keypress synergies late in training (**B**, “expert”), trial initiation 2–4 keypress synergies expressed relatively uniformly across training (**C**, “trial initiation”), synergies engaging variable number of keypresses that emerge and fade mid-training (**D**, “exploratory”), and early 1–2-keypress synergies largely confined to the first trial (**E**, “novice”).

Next, we quantified the emergence, decay, and overall prevalence of 1–5-keypress synergies at two timescales: within trials and across the full course of training (Fig. 4, representative single subject; Fig. 5, group data). Synergies were grouped by their percent usage across the session into four categories: <5%, 6–20%, 21–49%, and >50%. These bins were wide enough to ensure sufficient data within each category while providing a principled way to distinguish rare, moderate, and predominant synergies that may play different functional roles during learning.

Synergies used for <5% of training time appeared almost exclusively in the earliest trial(s), comprised 1–2 keypresses/synergy and then disappeared (green arrow in Fig. 4C; Fig. 5E). Synergies expressed for 6–20% of training time, emerged and faded mid-practice and likewise comprising 1–2 keypresses per synergy (dark blue arrow in Fig. 4C; Fig. 5D). Synergies expressed for 21–49% of training time included a type that occurred reliably at the start of trials as performance approached asymptote, persisted through session end, and encompassed 3–4 keypresses per synergy (red arrow in Fig. 4C; Fig 5C). Finally, synergies present for >50% of training time comprised the “predominant/expert” patterns, which arose mid-training, became dominant toward the end of practice, and could encompass up to 5 keypresses per synergy (purple arrow in Fig. 4C; Fig. 5B).

The most prevalent composition of the predominant expert multi-digit synergies observed in the last practice trial was “4–1–3–2” in 52% of subjects (Suppl Fig 4 and 5). The second most prevalent was “4–1–3–2–4” in 21% of *subjects*, the full trained sequence, which incorporate a repeated “4” spanning the sequence boundary (last keypress of one sequence followed by the first keypress of the next) to gain speed [41]. Next was “4–4-1” present in 16% of the subjects, also taking advantage of the 4–4 double tap. Finally, “4–4-1–3-2” was the predominant synergy in 11% of the subjects. Note that the last two expert synergy types crossed individual sequence boundaries. Together, these findings indicate that skilled performance in this skill task is supported by a relatively small repertoire of expert multi-digit synergies some of which exploit boundary double taps and possibly sequence fractionation (chunking) [42].

## Discussion

Here we show that complex, multi-digit synergies emerge rapidly after practice onset and increase progressively during the early phases of naturalistic skill learning, with their most prominent development occurring across intervening rest breaks. The synergies that supported acquisition of the trained skill were characterized by small, rapid, and overlapping digit movements that gradually crystallized over early trials and robustly predicted subsequent performance gains. Notably, these expert-like multi-digit synergies were insensitive to fatigue and remained stable into the following day.

### High-Order Multidigit Synergies Underlie Improvements in Naturalistic Skill

Motor learning—from everyday skills such as typing to highly trained behaviors like musical performance—relies on the production of temporally precise action sequences that form the basis of fine motor expertise [16, 42–44]. These sequences are not controlled muscle by muscle, but through coordinated motor synergies, making their reorganization central to skilled performance. Accordingly, examining how motor synergies reorganize with learning provides a direct window into the neuromotor processes that support the acquisition, refinement, and generalization of skilled actions.

To address this question, we recorded movements with a high-speed camera while subjects learned a new sequential motor skill. Markerless pose estimation software [45] and a dimensionality reduction strategy [45] allowed us to characterize synergies (unique combinations of covarying four-digit movements over one to five keypresses), as the new skill was learnt. Multi-digit synergies are the ultimate expression of efficient modular control strategies used by the central nervous system to execute sequential skills, like playing musical instruments [46–51]. We observed that synergy morphology evolved remarkably quickly, changing within seconds of training onset. Early practice rapidly sculpted the motor repertoire toward temporally compressed, overlapping, higher-order multi-digit synergies, whose stabilization and dominance from approximately the twelfth practice trial onward—when performance plateaued—robustly predicted the degree of skill acquisition and likely reflected an expansion of underlying memory capacity [26].

Synergies involving single digits, which predominated during the earliest trials, progressively transitioned into coordinated multi-digit patterns as training advanced. This rapid reorganization of synergy structure closely mirrored the time course of behavioral improvement. By the time performance approached a plateau, multi-digit synergies were preeminent and remained stable across an overnight interval, as assessed on Day 2, indicating the formation of a stable kinematic representation over time. This transition may parallel the rapid shift from stimulus-bound responding to internally generated sequence production, in which actions are guided by an internal memory representation rather than discrete external cues, as is typical of reaction-time–based paradigms [52]. The swift emergence of this internally driven mode of control suggests that developing motor kinematics, associated with an expanding skill memory [26], may become primary constraints on behavior.

### Mechanisms Underlying the Formation of Expert Synergies

How might multi-digit synergies emerge preferentially across rest rather than during practice? We found that behavioral improvements occurred primarily across the brief rest periods interleaved with practice (i.e., micro-offline gains), consistent with prior reports [15, 17, 25, 32, 53–55]. Micro-offline skill gains are up to four times larger than those identified following overnight sleep [15], are reproducible [55–59] and persist even when practice bouts are shortened to as little as 5 seconds, arguing against recovery from performance fatigue as their primary source [56]. It has been proposed that micro-offline gains represent a form of memory consolidation [15, 52, 60], a view strongly supported by mechanistic work. In humans, hippocampo-neocortical neural replay predicts micro-offline improvements [32] and intracranial EEG studies linked the density of hippocampal sharp-wave ripples (80–120 Hz)—canonical replay markers—to micro-offline gains during early learning [58, 59]. Moreover, perturbing neural activity during brief rest intervals akin to those observed in humans substantially reduces micro-offline gains in primates [60]. establishing a causal role for hippocampo–neocortical replay in early skill learning.

Our results substantially extend prior work by demonstrating that motor synergy maps can reorganize across rest periods, revealing a previously unrecognized neural–motor correlate of micro–offline learning. More broadly, our findings suggest that practice and rest make distinct yet complementary contributions to naturalistic skill learning. Rest intervals interleaved with practice may promote the binding of individual action components into higher-order synergies, effectively compressing the action space and freeing capacity for further learning [26]. In contrast, practice itself may primarily encode elemental action representations and provide the feedback necessary to optimize movement kinematics [61, 62].

Previous work posed a role of fatigue on performance dynamics during early skill learning [63, 64]. Although our design followed published guidelines to minimize it [40], accumulating fatigue with continued practice [39] could, in principle, still undermine the formation or stability of expert multi-digit synergies either within individual 10-s practice trials or cumulatively across the 36-trial session. We found no evidence this occurred. Once multi-digit synergies were established by the end of early learning, they remained stable, showing no decline within trials at the end of training (when fatigue would be maximal, Suppl. Fig. 3) or across the entire training session (Fig 2E). Furthermore, if fatigue had degraded synergy kinematics by the end of Day 1, we would expect a Day 2 rebound (i.e., increased multi-digit synergy use); instead, 4-keypress synergy expression remained comparable across days, with no evidence of rebound on Day 2. Thus, across three independent measures—and in contrast to prior assumptions [63, 64]—we found no evidence that performance of expert synergies during early practice of a challenging naturalistic skill are disrupted by motor or cognitive fatigue.

### Do Distinct Synergy Subtypes Subserve Early Naturalistic Learning?

Classifying synergies according to the proportion of training time in which they were expressed revealed distinct synergy subtypes. A set of synergies expressed for <5% of total training time (1–2 keypresses/synergy) appeared predominantly in the first few trials and then vanished (Fig. 4C, green arrow; Fig. 5E). These “novice” synergies likely reflect provisional kinematic solutions to the demands of a newly acquired skill, which are rapidly supplanted by higher-order, multi-digit synergies as learning unfolds. A second subgroup consisted of synergies expressed for 6–20% of training time (1–2 keypresses per synergy), which emerged and faded mid-practice (Fig. 4C, dark blue arrow; Fig. 5D). These “exploratory” synergies may reflect kinematic experimentation during the transition from stimulus-driven to internally generated execution of the naturalistic skill. Synergies expressed 21–49% of training time (1–4 keypresses per synergy) appeared consistently at trial onset as performance neared asymptote and remained present until the end of the session (Fig. 4C, red arrow; 5C). These “trial-initiation” synergies may reflect advance planning of the initial actions that structure the ensuing practice period [65].

Synergies expressed for >50% of total training time (up to five keypresses per synergy) constituted the “predominant/expert” class: they first appeared around mid-training and progressively came to dominate behavior toward the end of practice (Fig. 4C, purple arrow; Fig. 5B). The dominant features of these expert synergies were optimized speed–accuracy for repeated taps on the same key (or double taps) [41] and systematic fractionation of the trained sequence into preferred chunks [66–68]. This reorganization of skill synergies likely supports the early integration of fragmented, independent movements into highly coordinated, multi-digit actions, effectively reducing the functional degrees of freedom in expert performers [5, 6, 69].

The tight coupling between a small set of expert multi-digit synergies and high skill levels raises an intriguing translational question: could explicit training of these expert synergies complement conventional practice and accelerate learning in domains such as sport, musical performance, or post-stroke neurorehabilitation [70]? More broadly, a deeper understanding of the kinematic mechanisms that underlie skill acquisition may inform the design of more effective assistive and rehabilitative technologies, for example by guiding synergy-based control strategies in neuroprosthetics or robotics [7, 71].

### Limitations and Future Directions

Several limitations of the present work suggest fruitful avenues for future research. First, employing multi-camera motion capture would permit full three-dimensional kinematic reconstruction, enabling a more comprehensive characterization of movement structure than was possible here. Second, extending synergy analyses beyond distal digit segments to include additional digits and proximal effectors that support manual function—such as the wrist, arm, and shoulder—may uncover further kinematic contributions to skilled performance and clarify how distal and proximal control are integrated. Finally, future studies should test whether the kinematic mechanisms underlying sequence learning generalize to multi-limb coordination, postural control, and other forms of motor learning, an issue central to evaluating their broader relevance for neurorehabilitation of skilled function.

## Methods

### RESOURCE AVAILABILITY

Further information and requests for resources should be directed to and will be fulfilled by the lead contact, William D. Kistler (william.kistler.17@ucl.ac.uk).

### MATERIALS AVAILABILITY

This study did not generate new unique reagents or materials.

### DATA AND CODE AVAILABILITY

Data used in this study (subject to participant consent) will be made available upon reasonable request to the lead contact. All custom code used for processing, embedding, clustering, and statistical analysis is publicly available and archived at Zenodo [https://zenodo.org/records/10717889].

### EXPERIMENTAL MODEL AND SUBJECT DETAILS

#### PARTICIPANTS

Twenty right-handed, neurologically healthy adults (12 women; mean age = 29.2 ± 2.12 years, SD) participated under a protocol approved by the NIH Combined Neuroscience Institutional Review Board. Active musicians were excluded to avoid potential confounding effects on motor learning. Seventeen participants were excluded due to technical issues with video capture, yielding a final dataset of 20 participants. The target sample size was determined a priori by power analysis based on prior learning studies using the same task.

#### TASK

Participants performed a 5-digit sequence-typing task (41324) with their non-dominant (left) hand on a standard 104-key QWERTY keyboard. The little, ring, middle, and index fingers were mapped to numeric keys 1 through 4, respectively; the thumb was not used. Each trial consisted of 10 s of practice followed by 10 s of rest, and participants completed 36 such trials within a single session. Throughout practice, the target sequence was continuously displayed, and each keypress elicited an asterisk, providing real-time feedback on sequence progression but not on accuracy. During rest periods, the sequence was replaced by five “X” characters to discourage overt or covert rehearsal. The task was administered, and all responses were recorded, using PsyToolkit (www.psytoolkit.org).

#### DATA COLLECTION

Participants performed a 5-digit sequence-typing task (41324) with their non-dominant (left) hand on a standard 104-key QWERTY keyboard. The little, ring, middle, and index fingers were mapped to numeric keys 1 through 4, respectively; the thumb was not used. Each trial consisted of 10 s of practice followed by 10 s of rest, and participants completed 36 such trials within a single session. Throughout practice, the target sequence was continuously displayed, and each keypress elicited an asterisk, providing real-time feedback on sequence progression but not on accuracy. During rest periods, the sequence was replaced by five “X” characters to discourage overt or covert rehearsal. The task was administered, and all responses were recorded, using PsyToolkit (www.psytoolkit.org).

### DATA ANALYSIS

#### POSE ESTIMATION

Digit kinematics were captured at 120 frames/s using a high-speed camera positioned in front of the left hand (Fig. 1C, top). Markerless pose estimation [34, 35] was used to localize the distal interphalangeal joints of the four active fingers (Fig. 1C, top) [36], yielding approximately 43,000 frames of x–y pose data per participant over the full practice period (**STAR Methods**). The x coordinate primarily indexed lateral motion within the keyboard plane (adduction–abduction), whereas the y coordinate reflected vertical displacement perpendicular to the keyboard surface (flexion–extension; Fig. 1C–E, **STAR Methods**).

Pose estimation was implemented in DeepLabCut v2.2.2 (Python 3.7.12) with GPU acceleration via CUDA Toolkit 11.2 and cuDNN 8.2. Sixty frames per participant (1,200 total) were manually annotated for the x,y positions of all four fingers. A ResNet-50 backbone was trained for 400,000 iterations using DLC default settings (three color channels, pairwise terms enabled, unsupervised refinement disabled). For a single shuffle, training and test errors were 2.12 and 2.18 pixels, respectively (image resolution: 1280 × 720) (Suppl Fig. 6).

Framewise predictions with likelihood < 0.95 were flagged as low confidence and subjected to visual inspection. Missing samples were linearly interpolated when gaps were ≤ 3 consecutive frames; longer gaps were excluded from further analysis. No temporal smoothing or filtering was applied to the resulting trajectories prior to downstream analyses.

#### SKILL MEASUREMENT

Motor skill was operationalized as the number of correct sequences produced per second (cs/s). A sequence was scored as correct if it matched the canonical pattern (41324) or any of its circular permutations (e.g., 13241, 32414). For each 10 s trial, we computed the average instantaneous cs/s. To characterize learning over time, cs/s data were fit with an exponential growth function:

L(t)=C1+C2(1-e-λt)L(t)=C_1+C_2(1-e^[-λt])L(t)=C1+C2(1-e-λt)

where (C_1) denotes baseline performance, (C_2) the asymptotic learning gain, and (\lambda) the rate constant. Parameters were estimated using constrained nonlinear least-squares (trust-region-reflective algorithm). Cumulative learning was quantified by integrating (L(t)) over practice time and normalizing trial-wise gains by the total area under the curve. Across participants, 95% of total gains (period defining early learning) were achieved by Trial 12.

#### ONLINE AND OFFLINE LEARNING CONTRIBUTIONS

Microscale changes in skill performance were decomposed into online and offline components using correct sequences per second (cs/s) as the behavioral metric. Micro-online gains were defined as the within-trial change in cs/s, computed as the difference between the average performance in the first and last second of each 10-s practice trial. Micro-offline gains were defined as the between-trial change in cs/s, computed as the difference between the last second of a given trial and the first second of the subsequent trial. These measures were calculated over the first 12 trials, corresponding to the early learning phase. Cumulative micro-online and micro-offline changes were then summed across this interval to estimate their relative contributions to overall skill acquisition.

To characterize structural changes in motor behavior, we examined trial-level synergy distributions based on HDBSCAN-derived cluster labels assigned to each video frame. For each 10-s trial, we extracted the first and last 1-s windows (120 frames each at 120 fps) and quantified the composition of behavioral clusters (synergies) within each window as a normalized histogram, excluding frames labeled as noise. Jensen–Shannon divergence (JSD) was computed between the first and last second of each trial to index online synergy reorganization, and between the last second of one trial and the first second of the next to index offline reorganization. This approach enabled a direct comparison between kinematic reconfiguration and skill change at the microscale.

#### POSE TRANSFORMATION AND SYNERGY EXTRACTION

To incorporate temporal structure into the pose data, we applied Morlet wavelet transforms to the x- and y-coordinates of each digit at five frequencies (0.75, 1.45, 1.95, 2.35, and 2.75 Hz), chosen to span the range of rhythmic, voluntary finger movements observed in pilot recordings. This yielded a time–frequency representation for each frame in which each digit’s position was decomposed into oscillatory amplitudes at each frequency. The aim was to capture behavioral variation across multiple timescales and to allow for coordination between digits operating at potentially different frequencies—for example, distinct rhythmic patterns expressed by the pinky and index fingers within the same behavioral segment. The resulting feature vector for each frame comprised 40 dimensions (4 digits × 2 coordinates × 5 wavelets).

We then performed dimensionality reduction using Uniform Manifold Approximation and Projection (UMAP), as implemented in HUB-DT [72], with 15 nearest neighbors and a minimum distance of 0.1 to balance local continuity and global separation. This preserved meaningful structure in the data while enabling visualization and clustering of distinct motor behaviors in a reduced two-dimensional space.

#### CLUSTERING OF BEHAVIOURAL DATA

To identify motor synergies, we applied HDBSCAN (Hierarchical Density-Based Spatial Clustering of Applications with Noise) to the two-dimensional UMAP embedding of the wavelet-transformed pose data [72]. Each point in this embedding corresponded to a single video frame and thus captured both spatial and temporal structure. HDBSCAN grouped points into clusters based on local density (minimum cluster size = 200, minimum samples = 30), labeling sparsely populated regions as noise (label −1). Each resulting cluster was treated as a discrete behavioral unit, or motor synergy, whereas noise labels captured transitional or non-repetitive movements.

Unlike DBSCAN, which uses a single density threshold, HDBSCAN builds a hierarchy of clusters over multiple density levels and selects the most persistent solutions. This is well suited to behavioral datasets in which synergy patterns vary in frequency, duration, and consistency across trials or individuals[72]. The resulting labels provided frame-level, fully unsupervised classifications of movement, enabling objective segmentation of behavioral structure across training.

To characterize each synergy, we computed mean wavelet amplitude profiles across all frames within a cluster. These profiles summarized the dominant spatial and frequency features of each motor pattern, including digit-specific rhythmic contributions. Combined with the unsupervised clustering, this representation provided a principled basis for identifying, tracking, and comparing motor synergies over time.

For each trial, we computed the proportion of frames assigned to each synergy as a normalized histogram (summing to 1). Frames labelled as noise were excluded from this quantification but retained for visualization. These normalized synergy distributions served as the kinematics “fingerprint” of each trial and were used in all between-trial comparisons.

#### JENSEN-SHANNON DIVERGENCE AND PERMUTATION TESTING

To quantify how motor synergy distributions changed with learning, we computed the Jensen–Shannon divergence (JSD) between behavioral label distributions at different trial time points. JSD was chosen because it is symmetric, bounded, and robust to zero probabilities [73]—properties well suited for comparing sparse behavioral distributions. Unlike simpler distance metrics (e.g., Euclidean or cosine distance), JSD captures proportional changes across all labels in a scale-invariant and interpretable way, avoids directional bias, and uses smoothing that prevents divergence due to missing cluster labels.

For each participant, synergy labels within a trial were converted into a probability distribution by computing the relative frequency of each cluster. For each pair of trials, JSD was then calculated between these distributions to index changes in synergy composition. To assess whether observed JSD values exceeded chance, we used a nonparametric permutation test: cluster labels were randomly reassigned 100,000 times between the two distributions, recomputing JSD on each iteration to generate a null distribution. The empirical p-value was defined as the proportion of permuted JSD values greater than or equal to the observed value, preserving marginal structure while randomizing label correspondence.

For group-level inference, individual p-values were transformed to z-scores using the inverse standard normal distribution and combined across participants using Stouffer’s method, yielding a single aggregate z-statistic and Stouffer’s p-value. This framework—distributional comparison, permutation-based testing, and meta-analytic aggregation—provides a robust way to detect learning-related changes in behavioral strategy by quantifying how the overall weighting of synergies shifts as training progresses, rather than focusing on any single synergy in isolation.

#### CROSS-DAY GENERALIZATION

To assess generalization of motor synergies across days, we focused on the subset of participants who completed both Day 1 and Day 2 (n = 10). For each participant, pose estimation was performed jointly on Day 1 and Day 2 videos, yielding a single set of wavelet-transformed trajectories spanning both sessions (~86,000 frames per participant). A single UMAP embedding was then constructed from the combined dataset, providing a common low-dimensional space in which behavior from both days could be directly compared.

HDBSCAN clustering was applied once to this full embedding to identify recurring motor synergies independent of recording day. Each frame—whether from Day 1 or Day 2—was assigned a synergy label, enabling trial-level synergy distributions to be extracted simply by indexing the corresponding frame ranges. For instance, to examine generalization from the final trained trial (Trial 36, Day 1) to the first transfer trial (Trial 3, Day 2), we compared the synergy distributions of those specific epochs within the shared embedding.

As in earlier analyses, synergy distributions were normalized to probability distributions and compared using Jensen–Shannon divergence with permutation-based significance testing. This approach provided a coherent, within-subject measure of behavioral transfer across days in a unified representational space.

### QUANTIFICATION AND STATISTICAL ANALYSIS

All modeling and statistical analyses were conducted in Python 3.8+ using NumPy, SciPy, UMAP-learn, HDBSCAN, and scikit-learn. Exponential fits were obtained with scipy.optimize.curve_fit using boundary-constrained nonlinear least squares. JSD was computed using log-sum averaging, and permutation tests were implemented with custom Python code. Individual z-scores were combined at the group level using Stouffer’s method. Statistical significance was defined as α = 0.05. All analysis scripts and configuration files will be made available on Zenodo [https://zenodo.org/records/10717889].

## Supplementary Material

Supplement 1

## Figures and Tables

**Table 1:** Permutation Test Between Trials 1 and 12

Participant ID	Observed JSD	Permutation P-value	Z-score
001	0.8321	0.0228	2.2763
002	0.723	0.4831	0.7014
003	0.8305	0.0036	2.9147
004	0.7644	0.3933	0.8537
005	0.7864	0.1287	1.5191
006	0.8326	0.0226	2.2793
007	0.8016	0.3568	0.9215
008	0.8173	0.0641	1.8514
009	0.7972	0.6688	0.4277
010	0.8183	0.5271	0.6325
011	0.7709	0.4274	0.7936
012	0.8176	0.1939	1.2991
013	0.8186	0.2591	1.1285
014	0.7509	0.416	0.8134
015	0.8021	0.3161	1.0026
016	0.7057	0.5075	0.6627
017	0.8029	0.3199	0.9947
018	0.8298	0.0264	2.2195
019	0.8309	0.0269	2.2128
020	0.8272	0.03	2.1702
			Stouffer’s P: 6.083e-10

**Table 2:** Permutation Test Between Trials 12 and 36

Participant ID	Observed JSD	Permutation P-value	Z-score
001	0.8055	0.3724	0.8921
002	0.6119	0.8466	0.1935
003	0.4169	0.985	0.0188
004	0.4043	0.9645	0.0445
005	0.4633	0.9721	0.035
006	0.5681	0.7679	0.2951
007	0.4554	0.931	0.0866
008	0.4066	0.989	0.0138
009	0.8326	0.0112	2.5355
010	0.6235	0.8024	0.2502
011	0.3905	0.9668	0.0416
012	0.6548	0.7542	0.3131
013	0.7429	0.3663	0.9034
014	0.6653	0.6777	0.4157
015	0.7945	0.4672	0.727
016	0.3753	0.9754	0.0308
017	0.4596	0.8104	0.2399
018	0.3812	0.9785	0.027
019	0.4742	0.9174	0.1037
020	0.4269	0.9591	0.0512
			Stouffer’s P: 1.065e-01

**Table 3:** Permutation Test Between Trials 36, Day 1 and Trial 3, Day 2

Participant ID	Observed JSD	Permutation P-value	Z-score
001	0.3498	0.9868	0.0165
002	0.401	0.9683	0.0397
003	0.342	0.9973	0.0034
004	0.07	0.964	0.0451
005	0.0715	0.9685	0.0395
006	0.062	0.9693	0.0385
007	0.2415	0.9982	0.0022
008	0.4767	0.9278	0.0906
009	0.2129	0.9994	0.0008
010	0.2048	0.9849	0.019
			Stouffer’s P: 9.256e-01

**Key Resources Table:** 

REAGENT or RESOURCE	SOURCE	IDENTIFIER
Deposited data		
Video data	This paper	N/A
Software and algorithms		
MATLAB 2021b	The Mathworks, Natick, MA, USA	https://www.mathworks.com/
Python	Python 3.7.12	https://www.python.org/downloads/release/python-3712/
DeepLabCut	DeepLabCut 2.22	https://github.com/DeepLabCut/DeepLabCut
HUB-DT		https://github.com/Loken85/HUB_DT
Other		
NIH HPC Biowulf Cluster	NIH, Bethesda, MD, USA	https://hpc.nih.gov/
