# Supplementary material for: Expert Motor Synergies Emerge Predominantly Offline During Early Skill Learning
Source: bioRxiv. 2026 Feb 25:2026.02.24.707000. Preprint. [Version 1] doi: 10.64898/2026.02.24.707000 (PMC13119336; doi:10.64898/2026.02.24.707000)
Supplement: Supplement 1 [file NIHPP2026.02.24.707000v1-supplement-1.pdf]

bioRxiv preprint doi: https://doi.org/10.64898/2026.02.24.707000; this version posted February 25, 2026. The copyright holder for this preprint (which was not certified by peer review) is the author/funder. This article is a US Government work. It is not subject to copyright under 17 USC 105 and is also made available for use under a CC0 license.

# Supplementary figures

bioRxiv preprint doi: https://doi.org/10.64898/2026.02.24.707000; this version posted February 25, 2026. The copyright holder for this preprint (which was not certified by peer review) is the author/funder. This article is a US Government work. It is not subject to copyright under 17 USC 105 and is also made available for use under a CC0 license.

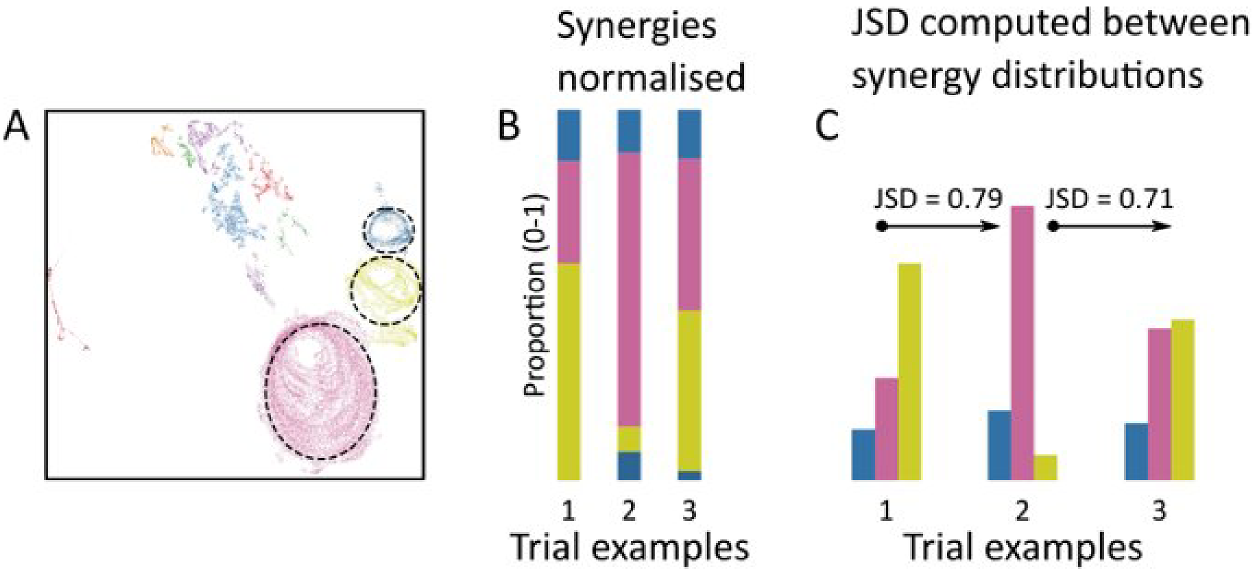


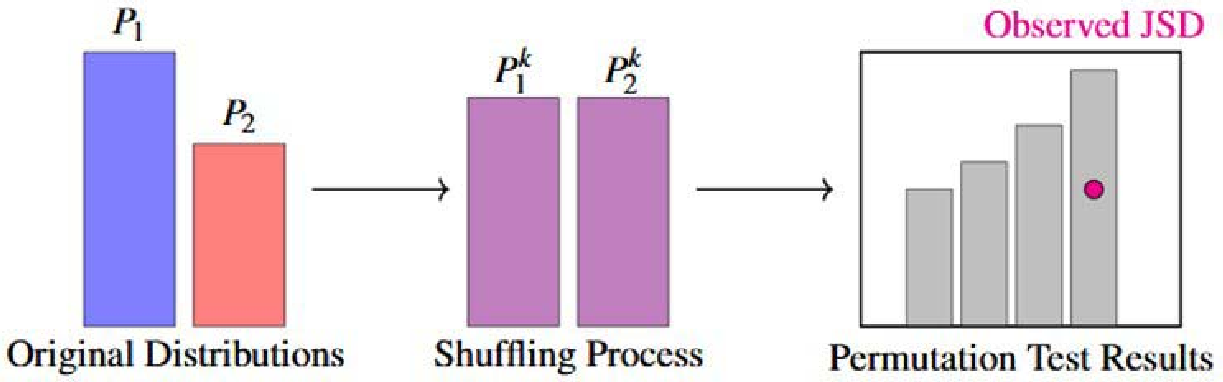


**SUPPLEMENTARY FIGURE 1:**

**(A)** UMAP embedding of an illustrative participant's behavioral data (same s in **Fig 1G**), highlighting selected multi-digit synergy clusters. **(B)** Normalized synergy distributions for three illustrative trials (noise removed). Each vertical bar depicts the proportional contribution of each synergy within a trial. **(C) Top**. Synergy data from (B) reorganized to enable direct comparison of synergy proportions across trials. Bars of the same color correspond to the same synergy label, and bar height indicates that synergy's proportion within a trial. When a synergy occurred multiple times in a trial, its contributions were summed (e.g., blue synergy in trials 2 and 3).

bioRxiv preprint doi: https://doi.org/10.64898/2026.02.24.707000; this version posted February 25, 2026. The copyright holder for this preprint (which was not certified by peer review) is the author/funder. This article is a US Government work. It is not subject to copyright under 17 USC 105 and is also made available for use under a CC0 license.

Jensen–Shannon divergence (JSD) values quantify differences between synergy distributions for each trial pair (e.g., trials 1 vs. 2, 2 vs. 3), providing a numerical index of behavioral reorganization during learning. **Bottom**. Conceptual overview of the permutation test used to assess the statistical significance of observed JSD values. Original synergy distributions (left) are randomly reassigned across synergy labels (center) to generate a null distribution of JSD values (right). The empirical JSD (pink dot) is then evaluated against this null distribution to determine whether the observed behavioral change exceeds chance.

bioRxiv preprint doi: https://doi.org/10.64898/2026.02.24.707000; this version posted February 25, 2026. The copyright holder for this preprint (which was not certified by peer review) is the author/funder. This article is a US Government work. It is not subject to copyright under 17 USC 105 and is also made available for use under a CC0 license.

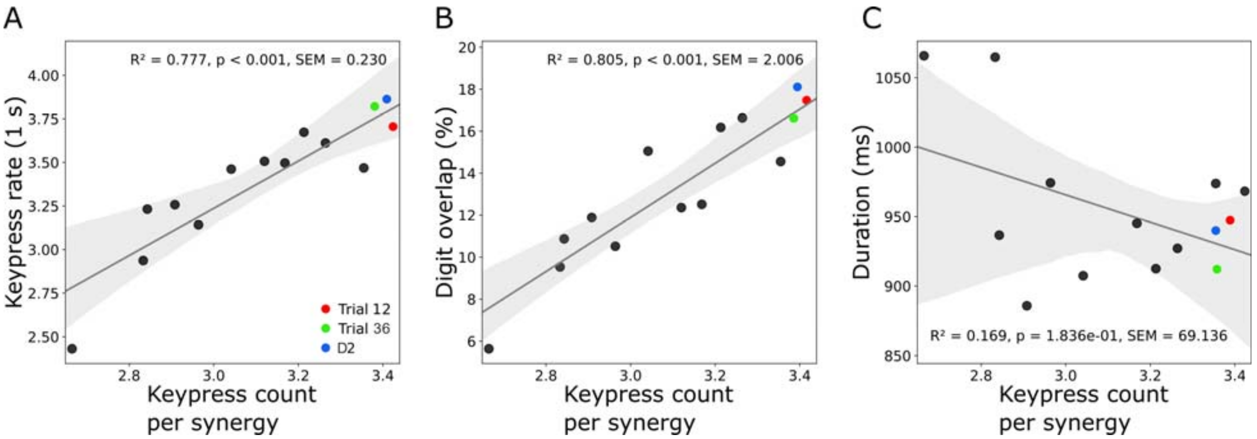


**SUPPLEMENTARY FIGURE 2. Relationship between the mean number of digits recruited per synergy and corresponding kinematics during early learning (trials 1–12).** The emergence of higher-order (multi-digit) synergies was associated with progressively faster keypress rates (A, $R^2 = 0.777$, $p < 0.001$) and increased temporal overlap of digit movements (B, $R^2 = 0.805$, $p < 0.001$), but not with changes in synergy duration (C, $R^2 = 0.169$, $p = 0.184$). Data points from Trial 36 (green) and Day 2 (b

bioRxiv preprint doi: https://doi.org/10.64898/2026.02.24.707000; this version posted February 25, 2026. The copyright holder for this preprint (which was not certified by peer review) is the author/funder. This article is a US Government work. It is not subject to copyright under 17 USC 105 and is also made available for use under a CC0 license.

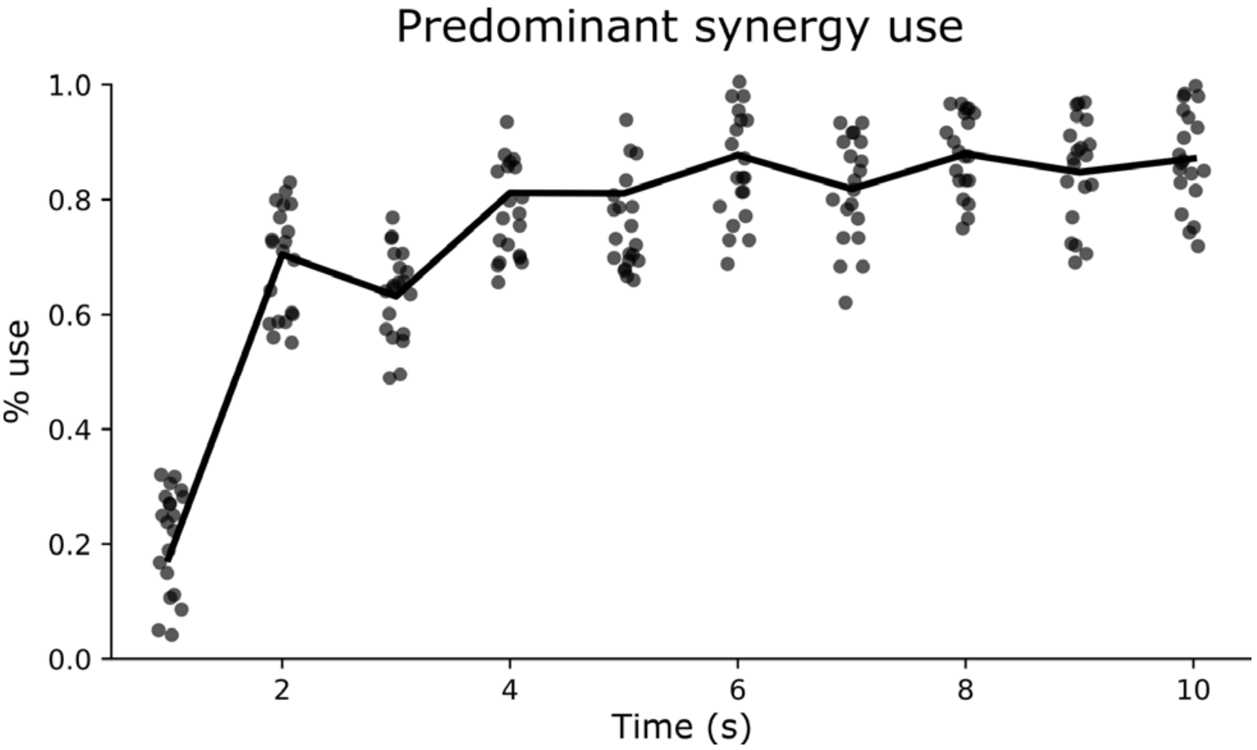


**SUPPLEMENTARY FIG 3. No sign of reduction in use of predominant multi-digit synergies late in training.**

We examined whether accumulating fatigue during the 10-s practice interval of a late trial (Trial 35)—at the end of Day 1, when fatigue would be most likely—reduced the percentage use of the predominant multi-digit synergies (see **Fig. 4**, **Fig. 5**). No such deterioration was observed: multi-digit synergies remained prominently and stably expressed throughout the 10-s interval of this late training trial in all participants. Thus, we found no evidence that accumulating fatigue measurably influenced intra-trial skill kinematics.

bioRxiv preprint doi: https://doi.org/10.64898/2026.02.24.707000; this version posted February 25, 2026. The copyright holder for this preprint (which was not certified by peer review) is the author/funder. This article is a US Government work. It is not subject to copyright under 17 USC 105 and is also made available for use under a CC0 license.

bioRxiv preprint doi: https://doi.org/10.64898/2026.02.24.707000; this version posted February 25, 2026. The copyright holder for this preprint (which was not certified by peer review) is the author/funder. This article is a US Government work. It is not subject to copyright under 17 USC 105 and is also made available for use under a CC0 license.

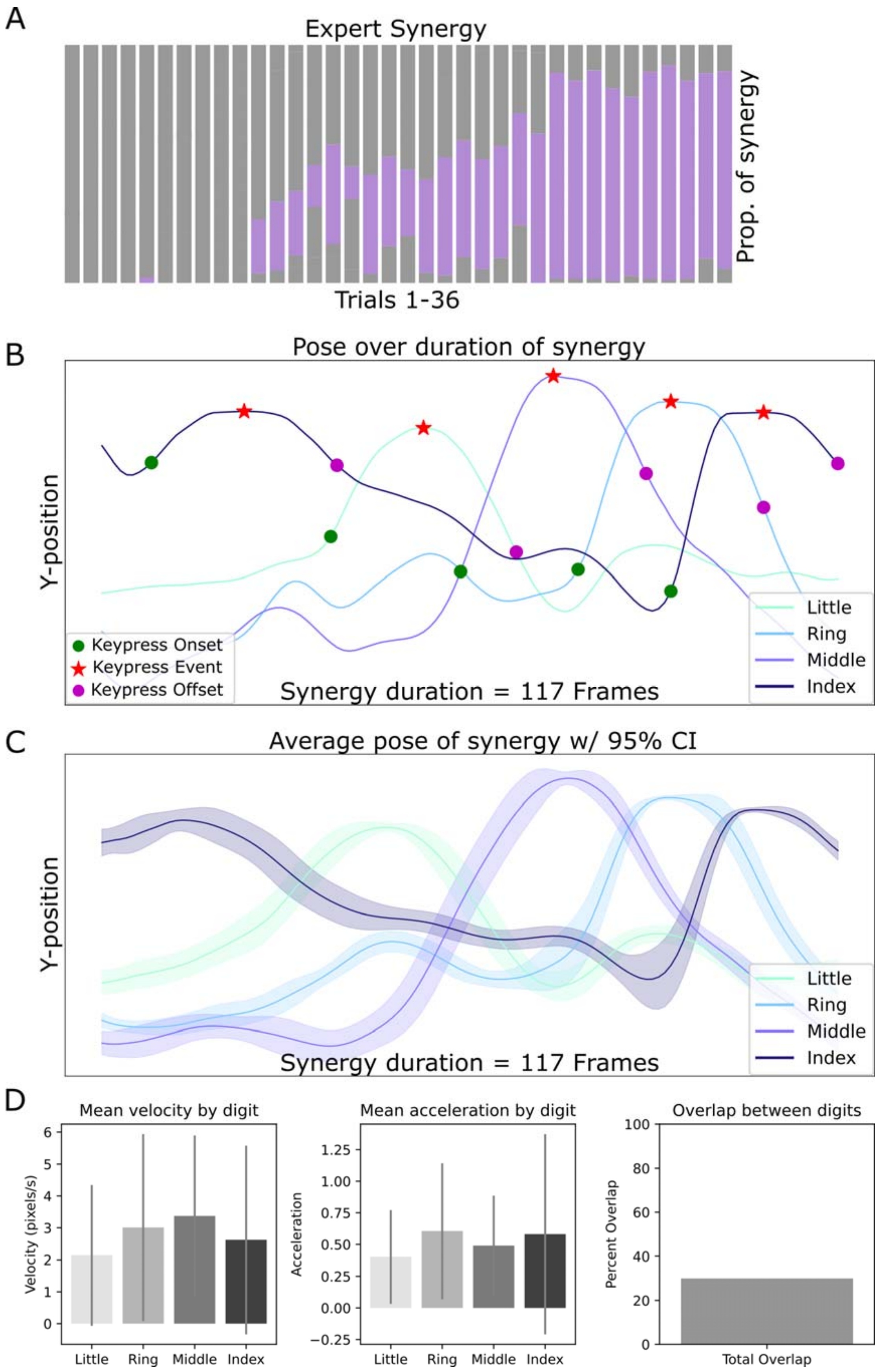


768

bioRxiv preprint doi: https://doi.org/10.64898/2026.02.24.707000; this version posted February 25, 2026. The copyright holder for this preprint (which was not certified by peer review) is the author/funder. This article is a US Government work. It is not subject to copyright under 17 USC 105 and is also made available for use under a CC0 license.

**SUPPLEMENTARY FIG 4**

**Kinematic Profile of an expert synergy (same subject as shown in Fig 4). (A)** A single expert synergy across 36 practice trials. Each vertical bar represents one trial, indicating the % use per trial of this synergy as training progressed. **(B)** Individual digit kinematics (Y-position, camera frame pixels) during execution of a single 5-keypress (red stars) expert synergy (117 frames, 975ms). **(C)** Mean digit trajectories of the same expert synergy averaged across all occurrences in the course of training (95% confidence intervals). The pattern highlights stable and consistent multi-digit coordination for the expert synergy once it is created. **(D)** Kinematic features of this expert synergy averaged in the course of training (±SDM): mean velocity (pixels/frame, left), acceleration (pixels/frame$^2$, middle) and digit overlap (% of frames with digit overlap in the course of training).

bioRxiv preprint doi: https://doi.org/10.64898/2026.02.24.707000; this version posted February 25, 2026. The copyright holder for this preprint (which was not certified by peer review) is the author/funder. This article is a US Government work. It is not subject to copyright under 17 USC 105 and is also made available for use under a CC0 license.

**Suppl Fig 5. Video reflecting expert synergy**

bioRxiv preprint doi: https://doi.org/10.64898/2026.02.24.707000; this version posted February 25, 2026. The copyright holder for this preprint (which was not certified by peer review) is the author/funder. This article is a US Government work. It is not subject to copyright under 17 USC 105 and is also made available for use under a CC0 license.

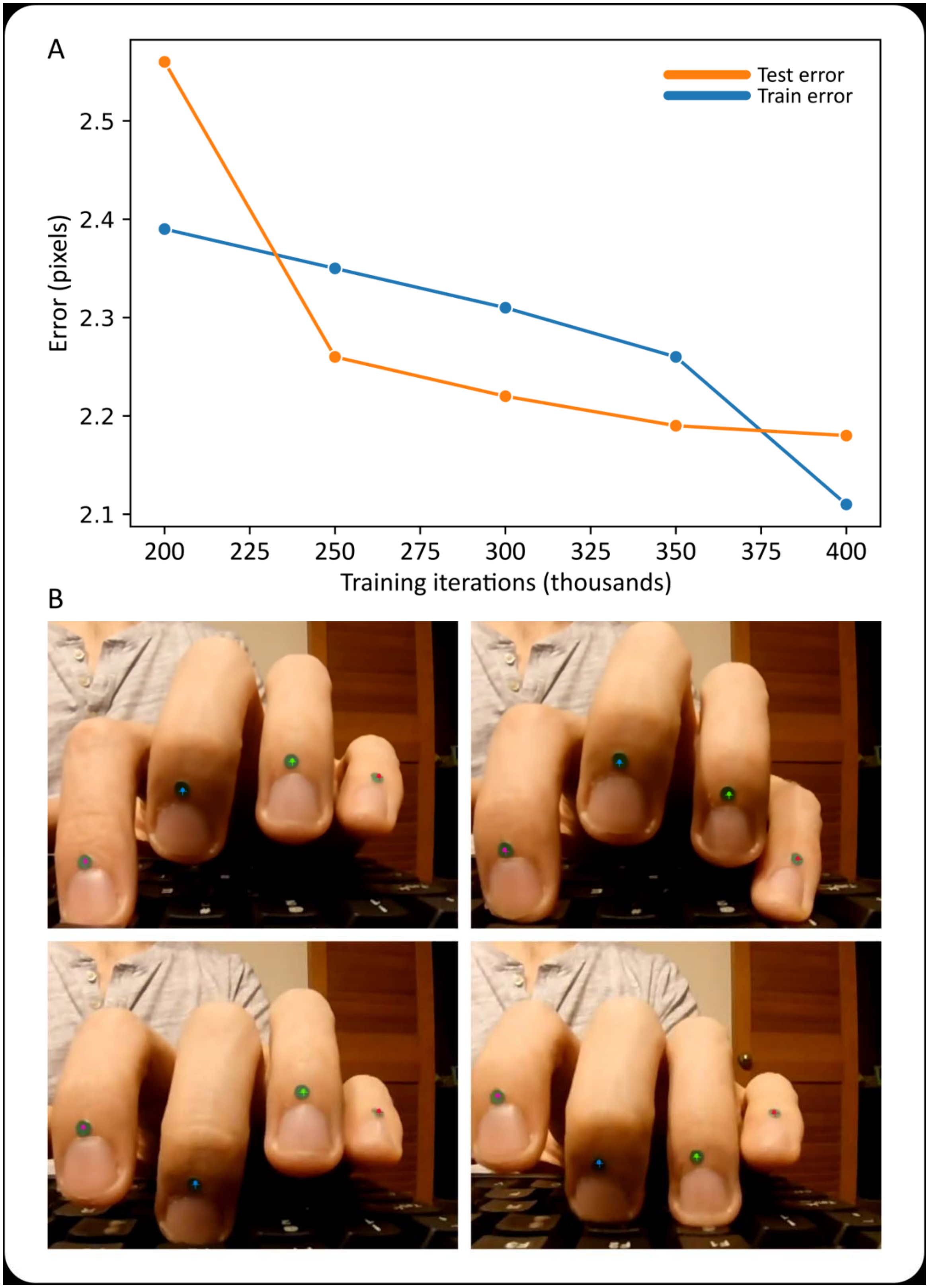

bioRxiv preprint doi: https://doi.org/10.64898/2026.02.24.707000; this version posted February 25, 2026. The copyright holder for this preprint (which was not certified by peer review) is the author/funder. This article is a US Government work. It is not subject to copyright under 17 USC 105 and is also made available for use under a CC0 license.

**SUPPLEMENTARY FIG 6**

**DeepLabCut network training performance and example marker labelling. (A)** A DeepLabCut neural network was trained to track digit movements using default parameters (400,000 training iterations, RGB input, pairwise terms enabled, without additional supervision). Network performance was evaluated using 1 shuffle split. Root Mean Square Error (RMSE) decreased steadily with training iterations, reaching 2.12 pixels on the training set and 2.18 pixels on the held-out test set (image resolution: 1280 × 720). Error was assessed at intervals of 25,000 iterations. The stability and convergence of both training and test errors indicate that the algorithm learned successfully. **(B)** Representative video frames showing predicted marker placements (coloured dots) on the fingernails of the index, middle, ring, and little fingers. Each marker corresponds to a tracked key anatomical point manually defined during training. Marker predictions shown here were generated by the trained DeepLabCut model and used as input for downstream kinematic analyses of digit position and motion.

During the preparation of this work the authors used GPT-5.2 for editing purposes. After using this tool/service, the authors reviewed and edited the content as needed and take full responsibility for the content of the published article.
